# Distal Displacement of Maxillary Sinus Anterior Wall Versus Conventional Sinus Lift with Lateral Access: A 3-Year Retrospective Computerized Tomography Study

**DOI:** 10.3390/ijerph17197199

**Published:** 2020-10-01

**Authors:** Giovanni Battista Menchini-Fabris, Paolo Toti, Giovanni Crespi, Ugo Covani, Roberto Crespi

**Affiliations:** 1 Department of Multidisciplinary Regenerative Research, Guglielmo Marconi University, Via Vittoria Colonna, 11, 00193 Rome, Italy; gb.menchinifabris@gmail.com (G.B.M.-F.); robcresp@libero.it (R.C.); 2 San Rossore Dental Unit, Viale delle Cascine 152 San Rossore, 56122 Pisa, Italy; 3 Department of Stomatology, Tuscan Stomatological Institute, Foundation for Dental Clinic, Research and Continuing Education, Via Padre Ignazio da Carrara 39, 55042 Forte Dei Marmi, Italy; gio.crespi@hotmail.it (G.C.); covani@covani.it (U.C.)

**Keywords:** dental implant, maxillary sinus, bone augmentation, CT imaging, infracture approach

## Abstract

Background: The present study is designed to compare the outcomes of two sinus augmentation procedures: distal displacement of the anterior wall versus standard sinus lifting and grafting with a lateral window approach. Methods: In the displacement group, a localized surgical fracture of the sinus floor achieved through an electromagnetic device results in the distal displacement of the anterior wall. In the filling group, sinus lifting (with lateral access) and grafting with particulate xenogeneic bone substitute was performed. Bone volume beneath the maxillary sinus was investigated with computerized tomography after baseline and postoperative data superimposition. Clinical and radiological outcomes over three years had been evaluated. Results: Forty-three dental implants were selected. The two sinus lift procedures significantly increased the bone volume (*p*-value ≤ 0.0017) in the displacement group from 1.17 ± 0.34 to 1.53 ± 0.39 cc, with a final bone gain of +0.36 ± 0.17 cc, and in the filling group from 1.24 ± 0.41 to 1.94 ± 0.68 cc, with a bone augmentation of +0.71 ± 0.31 cc. No events of dental implant bulging into the maxillary sinus occurred. Two implants failed early on in the filling group, attesting the 3-year survival rate of 92.6% (CI95%: 82.7–100%). Marginal bone loss at the distal aspect was 1.66 ± 0.72 and 1.25 ± 0.78 mm, respectively, for the displacement and filling groups, with a significant difference (*p*-value = 0.0497). Conclusion: Results showed a significant and effective bone gain around dental implants at a 3-year survey for both sinus augmented by backward displacement of the anterior wall (+34%) and sinus lifting and grafting with a lateral window approach (+57%).

## 1. Introduction

Maxillary sinuses are two-side cavities within the splanchnocranium above the posterior maxillary area of the alveolar bone. The dimensions of these pyramidal cavities are extremely variable from person to person and depend on peculiar interior features, that is, the presence of congenital or secondary sinus septa. A secondary septum is generally caused by loss of the alveolar bone surrounding root apices of an extracted tooth, which contributes to maxillary sinus hyperpneumatization [1]. Since standard-length implants cannot be placed in cases of severe bone deficiencies, different techniques of sinus augmentation are described in the literature to solve the problem of excessive pneumatization observed in the internal aspect of the floor of the maxillary sinus [2]. All the bone reconstruction procedures of the maxillary sinus are used to increase the existing height of the residual bone; even though they are generally considered inevitable, impacts on the Schneiderian membrane integrity and severe complications may occur [3]. Implant placement in the posterior maxilla is particularly challenging when compared to other areas since iatrogenic sinus membrane rupture is a commonly encountered complication, especially when the selected implant length is more than the available bone height. This has been identified as a well-documented cause of implant failure in the posterior upper jaw [4].

Boyne and James suggested a lateral approach to the maxillary sinus floor [5]. A bone window was opened through the lateral wall; then, autogenous bone marrow harvested from the iliac crest was grafted into the sinus cavity. When there was enough bone for primary stability, implants were placed simultaneously. On the other hand, they were placed at a later stage when graft healing was achieved. In case the height of the residual alveolar crest is less than 4 mm [6,7], a two-stage procedure may be appropriate. Several studies have evaluated the clinical and radiological outcomes in patients who underwent transcrestal sinus floor elevation (TSFE) [8,9,10]; they allow manipulation of the bone through the alveolar ridge to conserve osseous tissue, to increase residual alveolar bone height, and to improve density around dental implants placed in soft maxillary bone with a successful loading; successive evolutions of the TSFE intended to perform an indirect, simultaneous, osteotome-mediated sinus elevation procedure, with or without the use of bone substitute materials [11,12].

Regarding the possible rehabilitation strategies without the need for bone augmentation, short implants (less than 6 mm) can be successfully loaded in maxillary bone with a residual height from 4 to 6 mm, but their long-term prognosis is still questionable [13].

The primary aim of the present study is to evaluate, at a 3-year follow-up, the effect of different sinus lifting approaches (distal displacement procedure of the maxillary sinus anterior wall or standard sinus lifting and grafting surgery with a lateral window approach) on the volume remodeling of the crestal bone around implant-supported fixed prostheses. The secondary aim is to assess, at a 3-year follow-up, the presence of differences between the two sinus augmentation groups on implant survival and peri-implant marginal bone loss.

## 2. Materials and Methods

### 2.1. Patient Selection

A set of patients among consecutive subjects treated at the Tuscan Stomatologic Institute between February 2012 and April 2015 were retrospectively selected for the present study. Such patients had been followed-up for 3 years after surgery (2012–2017) at the Complex Operating Unit of Maxillo-Facial Surgery of the University of Pisa. Clinical and radiological information was collected. The preparation of the manuscript followed the STROBE statement. All procedures performed in studies involving human participants are in accordance with the ethical standards of the institutional and/or national research committee and with the 1964 Helsinki declaration and its later amendments or comparable ethical standards. (Ethical Committee for retrospective analysis 2626-2008 PROT n° 58183).

### 2.2. Inclusion Criteria

- sinus lifting, with either backward displacement of the anterior wall or with lateral access, and grafting with particulate bovine graft;
- dental implants placed in the augmented sinus very close to the native anterior wall (premolar area);
- preoperative and postoperative maxillary computerized tomographic scans.
- Patients were excluded if any of the following information was in their medical record:
- no loaded implant during the 3-year follow-up;
- lack of postoperative radiographic 3-dimensional (3D) data up to 3 years after augmentation;
- preoperative bone thickness between the sinus floor and the edentulous crest of less than 3 mm;
- patients without chronic systemic diseases;
- excessive smoking habits (>10 cigarettes a day);
- alcohol or drug abuse;
- patients unwilling or unable to cooperate in maintaining oral hygiene and following medical prescriptions.

All the surgical interventions were performed by a single surgeon; moreover, a single prosthodontist was responsible for providing all prosthetic treatments.

### 2.3. Surgical Procedures

Proper premedication with antibiotics was given within one hour before surgery (2 g of amoxicillin or clindamycin 600 mg if allergic to penicillin). Then, the same drug was administered (1 g amoxicillin or 300 mg clindamycin if allergic to penicillin) twice daily for six days. In the sinus wall displacement group (displacement), after treatment with local anesthetic (optocaine 20 mg/mL with adrenaline 1:80.000, Molteni Dental, Scandicci, FI, Italy), a partial-thickness flap was raised to preserve the periosteum. The present management of soft tissues (mainly the intactness of the periosteum) is crucial to maintain the integrity of the blood supply and to promote healing by the secondary intention of tissues surrounding the implant site [14].

The primary incision was beveled and slightly palatal to vestibularly displace the keratinized residual tissue. Then, preservation of the papillae was accomplished by making releasing incisions a few millimeters from the residual teeth.

**Figure 1:**
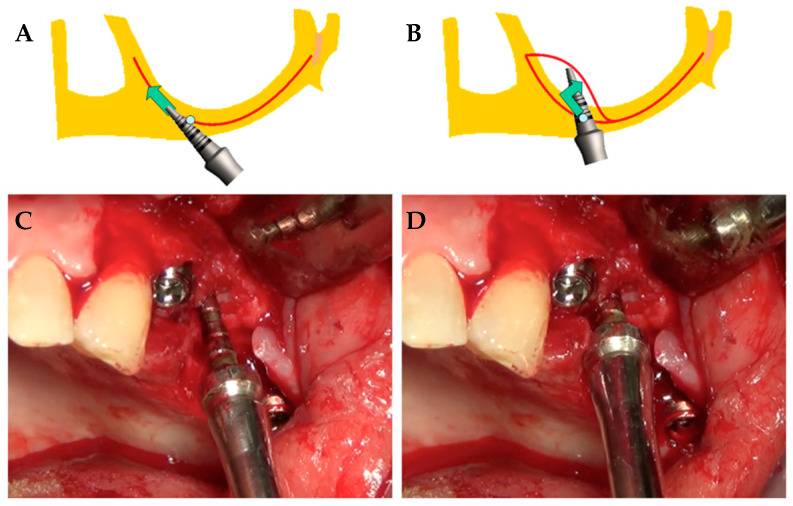
Augmented sinus by backward displacement technique of the anterior wall: (**A**,**B**) Procedure scheme for the surgical treatment; (**C**,**D**) clinical photograph showing the edentulous ridge of the maxilla with expansion devices in action.

A vertical fissure was opened within and through the residual alveolar bone with a blade mounted on an electromagnetic device (Magnetic Mallet, www.osseotouch.com, Turbigo, Milano, Italy) [15,16]. Such a device was drawn along the crest of the ridge, through the periosteum, cortex, and spongiosa, and towards the floor and anterior wall of the maxillary sinus, where the prosthetic implant palatal emergency was planned (Figure 1A). Rounded tips were used to displace the periosteum-free hard tissue mass within the sinus in the posterior-palatal direction along the side of pristine residual bone volume. This procedure allows clinicians to distally push the anterior wall of the sinus, following a parallel direction to the pristine palatal vault. The ultimate result of dislocation was the creation of new space between the two lateral walls and the mesiodistal as well (Figure 1B). 

The implant site was created, both distally against the preexisting lateral walls and apically, moving up and compressing with a progressive increasing diameter of bone expanders (Figure 1C,D). Reorientation towards the ideal (verticalized) prosthetic axis was then obtained by gradually extracting the instruments by forcing the tips of the instruments during removal. The final cavity should remain underdimensioned in both height and width so that the final plunge is produced by the implant itself, which will be stabilized in the native bone available under the floor of the sinus.

External-hexagon, cylindrical-shaped body, flat-grooved apex, sand-blasted, and acid-treated surface osseointegrated pure titanium grade 4 dental implants (Pro-Link^®®^ Out-link, Sweden & Martina, Due Carrare, PD Italia) were placed.

The flaps were firmly sutured to the mucosa and the periosteum, with holding sutures stabilizing the collagen material (Gingistat, Acteon Pharma, Bordeaux, France) to control the bleeding and to ensure blood clot stability. After 90 days of healing, implants were loaded.

**Figure 2:**
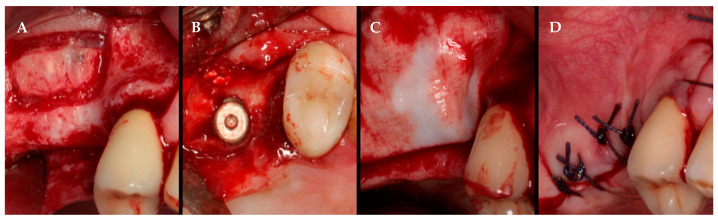
Augmented sinus by lifting and grafting with a particulate bone substitute with a lateral window approach: (**A**) Exposure of the crest with full-thickness flap with osteotomies; (**B**) dental implants placed in sinus lifted and grafted with xenogeneic bone substitute material; (**C**) surgical site covered by reabsorbable membrane; (**D**) clinical view of surgical procedure with final sutures of gingival margins.

In the group in which sinus was lifted with a particulate bovine bone graft using the lateral access technique (filling), after treatment with local anesthetic (optocaine 20 mg/mL with adrenaline 1:80.000, Molteni Dental, Scandicci, FI, Italy), a lateral wall was fenestrated and an inferior horizontal osteotomy line was positioned, beveled at the sinus floor level; anterior and posterior vertical osteotomies were performed 5 mm outside of the borders of the location in which the dental implant had to be placed (Figure 2A). Using a sinus membrane elevator, the sinus membrane was gently separated from the sinus floor and the lateral wall was removed. Implants were placed according to the manufacturer’s protocol. The sinus cavity was grafted using 100% particulate deproteinized anorganic bovine bone graft (Bio-Oss^®®^, 0.5–1 μm particle-size, Geistlich Biomaterials Italia srl, Thiene, VI, Italy) mixed with blood (Figure 2B). Then, the vestibular wall was covered by a resorbable collagen membrane, and flap closure was completed using silk interrupted sutures (Figure 2C,D). The sutures were removed 7 days after the surgery. After 90 days of healing, implants were loaded.

### 2.4. Clinical Variables

Patients were screened, and complications that arose during implant/prosthesis maintenance care from biological (implant failure), technical (repaired or replaced prosthesis), and mechanical (fracture of the metal components, framework, or structures) point of view were recorded in their case-sheets.

If experienced pain were reported in the case-sheet or if the patient showed any signs and symptoms of infection of the soft tissues surrounding the implants, the dental fixture was individually tested (in the case of a multiple-implant prosthesis design, the prosthesis was removed). An implant was classified as failing on the day of its removal for one of the following conditions: implant fracture; the presence of implant mobility and/or pain/discomfort/neurologic disorder [17], spontaneous or stimulated, after applying a force with two metallic handles of dental instruments; the presence of persistent inflammation or chronic suppuration, and apparent radiographic bone loss (greater than 80% in depth along implant direction) [18]. Implant survival rate, following criteria described by Anusavice [19], with a dichotomous grade of adverse effects, was calculated according to Romeo and coworkers [20].

### 2.5. Radiographic Assessments

**Figure 3:**
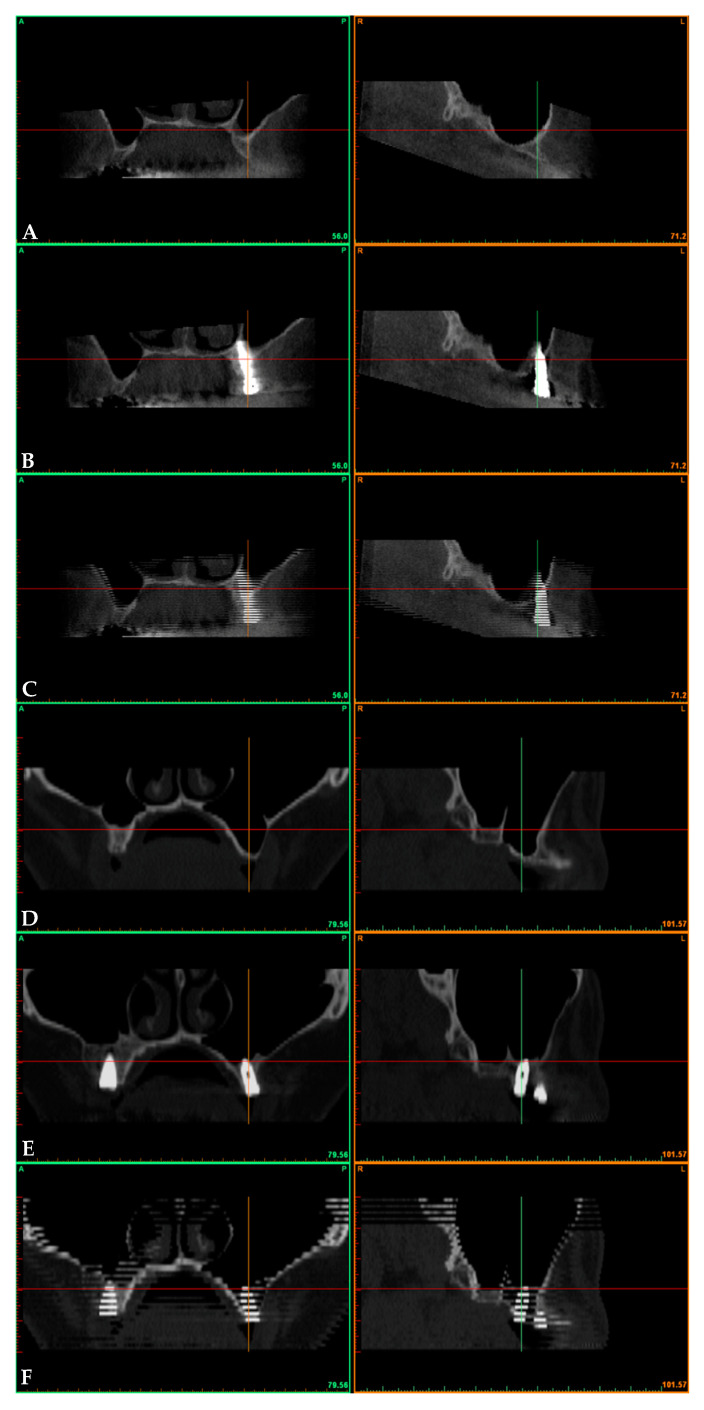
Fontal and lateral views of an augmented sinus by a backward displacement technique of the anterior wall: (**A**) preoperative; (**B**) postoperative; (**C**) fused files. Fontal and lateral views of an augmented sinus by lifting and grafting with a particulate bone substitute with a lateral window approach: (**D**) preoperative; (**E**) postoperative; (**F**) fused files.

The CT scans were preoperative (preop) and postoperative at 3 years after prosthetic loading (3yrs). Preoperative and postoperative CT scans were superimposed according to Crespi and coworkers in cases of both the displacement (Figure 3A–C) and filling groups (Figure 3D–F) [21]. Then, superimposed data have been saved as a file with DICOM extension (Digital Imaging and Communications in Medicine) [22]. Once data had been processed, volumes (V) were measured as per Sbordone [23] within a standardized volume of interest (VOI) contained within the following boundaries: 10 mm mesially and 10mm distally, 10 mm buccally and 10mm palatally to the center of implant shoulder.

In CT sections, the following variables were assessed: bone volume (BV) before sinus surgery (preop), 3 years after surgery (3yrs) and their difference (ΔBV preop→3yrs, from Equation (1)) or fractional gain (in percentage, from Equation (2)):

(1) $$\Delta \mathrm{BV}={\mathrm{BV}}_{3\mathrm{yrs}}-{\mathrm{BV}}_{\mathrm{preop}}$$

(2) $$\%\Delta \mathrm{BV}=100\cdot \frac{{\mathrm{BV}}_{3\mathrm{yrs}}-{\mathrm{BV}}_{\mathrm{preop}}}{{\mathrm{BV}}_{\mathrm{preop}}}$$

Marginal bone level (MBL) was evaluated on radiographic cross-sectional images 3 years after surgery. MBL is the distance between the fixture–abutment interface and the most apical point of the bone-to-implant contact. Dental implants were inserted, as recommended, at the crestal bone level, so MBL was assumed to be close to zero at baseline (just after surgery). Marginal bone loss (ΔMBL), which could be obtained from Equation (3), was approximated to the 3-year marginal bone level. Changes at the mesial and distal ΔMBLs were averaged.

(3) $$\Delta \mathrm{MBL}={\mathrm{MBL}}_{3\mathrm{yrs}}-{\mathrm{MBL}}_{\mathrm{baseline}}≅{\mathrm{MBL}}_{3\mathrm{yrs}}$$

The angle of displacement in degrees between the pristine sinus floor and the displaced bone plate at a 3-year survey was determined by using a dentascan software program from a frontal view, passing through the center of the dental implant.

### 2.6. Statistical Analysis

Statistical analyses were performed using a statistical tool package (Statistics Toolbox, MatLab 7.11; The MathWorks, Natick, MA, USA). The Shapiro–Wilk test did not confirm the normal distribution of the outcomes’ data for all the subgroups investigated (Table 1). Sample sizes were calculated with power = 80%, α = 0.05, and β = 0.20. For a more conservative analysis of pair-wise comparisons, significant differences between times (matched data) were assessed by the Wilcoxon signed-rank test, whereas significant differences between groups (independent data) were identified by the Wilcoxon rank-sum test. The level of statistical significance was set at 0.05.

## 3. Result

### 3.1. Results

Fifty-six patients were originally included in the study (29 in the displacement group and 27 in the filling group). Out of 56 included implants, two (both belonging to the filling group) failed early on. Following the exclusion criteria, finally, 43 patients were selected for further analyses (18 males and 25 females), with a mean age of 56.3 ± 9.0 years and a total of 43 implants enrolled.

### 3.2. Surgical and Prosthetic Findings

At the 3-year survey, the resulting implant survival rate was 100% for the displacement group, whereas the 3-year survival rate for the filling group was 92.6% (CI95%: from 82.7% to 100%). In the displacement group, one out of 23 sinus lift procedures resulted in the perforation of the membrane at the moment of the surgery. A single event of postoperative nasal bleeding was registered (in a male patient), and no associated pain or mobility of the dental implant was recorded. Few episodes of minor swelling were reported for both groups during the first days of healing, but neither flap dehiscence, nor mucositis, or suppuration was observed. For both groups, the implants were provisionally loaded within 90 days after placement. In the displacement group, the final ceramic restorations were delivered within 17 weeks after surgery, whilst in the filling group, final restoration was functionally placed within 36 weeks from augmentation surgery.

### 3.3. Radiological Evaluation

**Figure 4:**
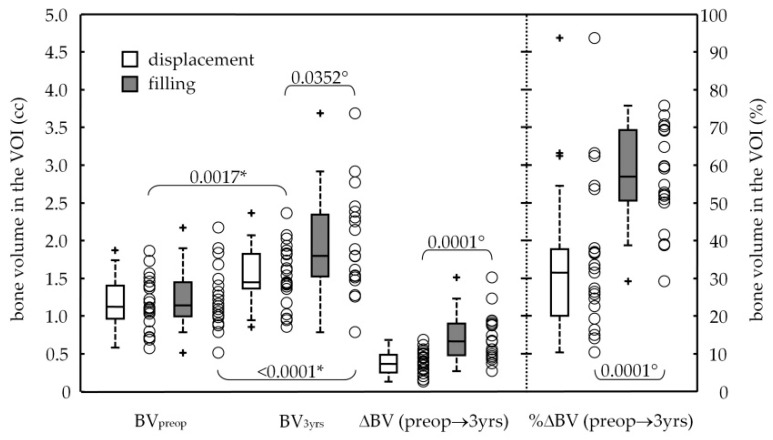
Box and wishers plot (with scattering data, O) of the volume at the preoperative stage (BVpreop) and at the 3-year survey (BV3yrs) of alveolar bone beneath the sinus, and their difference, ΔBV (with its percentage, %ΔBV) from the preoperative stage to the 3-year survey (preop→3yrs) in volume of interest (VOI). In box-and-whiskers plot the box line represents the lower, median and upper quartile values, the whisker lines include the rest of the data. Outliers (+) were data with values beyond the ends of the whiskers. Results for pair-wise statistical comparisons: Wilcoxon signed-rank test assessing changes in time from the preoperative stage to the 3-year follow-up (*); Wilcoxon rank-sum test assessing changes between groups (°).4. Discussion.

Radiographic volumes of the bone beneath the sinus were measured in the VOI before and about three years after sinus augmentation (Table 1 and Figure 4). Significant differences were recorded between times showing gains in bone volume of 0.36 ± 0.17 and 0.71 ± 0.31 cc for the displacement and filling groups, respectively. The net increase in bone resulted by measuring the volume of the alveolar bone beneath the displaced sinus in the VOI from the preoperative time (1.17 ± 0.34 cc) to the 3-year follow-up (1.53 ± 0.39 cc), with a *p*-value of 0.0017; again, the bone beneath the maxillary filled sinus within the VOI increased from 1.24 ± 0.41 to 1.94 ± 0.68 cc, with a significant difference (*p*-value < 0.0001). No events of dental implant bulging into the maxillary sinus had occurred at the 3-year survey. In terms of percentage increase of bone volume after the backward distraction of the anterolateral portion of the sinus floor (Δ%), it was 34 ± 21%. The sinus lifting and grafting with particulate bovine material allowed a bone gain of 57 ± 13%.

Marginal bone loss around fixtures registered at 3 years after implant placement was 1.47 ± 0.38 and 1.30 ± 0.58 mm for the displacement and filling groups, respectively (Table 1). No significance between the two groups was registered except for the distal aspect of the implant, in which linear bone loss was 1.66 ± 0.72 mm for the displacement group and 1.25 ± 0.78 mm for the filling group (with *p*-value = 0.0497).

The rationale of the study was to report the middle-term effectiveness of the distal displacement of the maxillary sinus anterior wall by using electromagnetic devices and osteotomes in patients with a bone thickness beneath sinus close to 3 mm (that is, a class D sinus) [6].

Even though the use of a tilted dental implant and short/ultrashort implant beneath the sinus seemed to guarantee an adequate clinical performance, when the bone height was judged insufficient for the abovementioned rehabilitation strategies, augmentation procedures were required [24]. The osteotomy technique seemed to be adequate for elevating the Schneiderian membrane when the residual bone height was at least 5 mm. When the bone loss was more accentuated, a lateral antrostomy was generally recommended for placing fixtures with adequate length [25]. However, bone augmentation in the horizontal and vertical directions could be the solution when the pristine bone was not adequate for standard implants, with or without the use of bone substitute materials.

After the creation of a “greenstick” fracture malleting the pristine maxillary sinus floor, the clinician compressed the cancellous bone within the osteotomy site up and backward to increase the amount of volume bone. The radiographic analysis reported an increase of the available bone with a mean bone gain of 0.36 cc, being the bone around the dental implant that had been stable for at least three years. The data were compared with outcomes of well-established sinus augmentation procedures (that is, sinus lifting and grafting with particulate bovine graft with lateral access), a mean bone gain of 0.71 cc was registered 3 years after augmentation surgery. It appeared that the increase of bone volume in the displacement group was about half of that from the standard sinus lift and grafting technique. However, this was because the clinician preferred to increase, as much as possible, the space between the sinus floor and the elevated Schneiderian membrane after the patient underwent bone lid technique. 

A modified double bone lid technique could be performed when the alveolar antral artery is clearly observable in the buccal wall of the maxillary sinus and it is considered safe and predictable. However, when applicable, distal displacement of the maxillary sinus anterior wall is intended to reduce bleeding in the alveolar antral vascular plexus and all the other potential complications of surgical vascular injury [26]. The backward displacement of the anterior sinus wall seemed to be a less-demanding procedure to allow sufficient bone volume to properly support a dental implant placed in an edentulous bicuspid area.

The only study describing a volume gain in the atrophic maxillas after sinus lift augmentation with a bovine-bone-substitute material was the case series of Scarano in which the author reported a volume ranging from 1.40 to 2.81 cc at a 6-month follow-up [27]; the values were in line with outcomes of the present paper.

To the best of our knowledge, there seemed to be no data regarding the results of three-dimensional volume change measurements of techniques similar to that described here; moreover, when healing of a corticocancellous block bone grafted into the maxillary sinus was investigated, a repneumatization phenomenon was reported between the first and second year. The graft resorption was close to 50%, with a mean volume of the grafted bone of 0.66 cc; moreover, their procedure was a more demanding surgery than the displacement procedure that was described here [28].

While some studies attested a mean linear bone gain in the range of 5.67–10.9 mm after maxillary sinus lift with a membrane elevation procedure [29,30,31], several other authors experienced, with the osteotome technique, a bone gain lower than the abovementioned gain, ranging from 1.8 to 3.94 mm [32,33,34,35,36]. Even if the technique did not appear to be similar to those described here, outcomes confirmed that the sinus lift carried out with a lateral window approach led to a higher increase of bone volume than that obtained for the osteotome-mediated transcrestal sinus lift approach. Furthermore, to prevent intraoperative complications such as alveolar ridge fracture, tooth damage, and hemorrhagic events, less surgically demanding procedures (such as piezoelectric devices or manual bone scrapers) have been used to prepare the lateral window and to separate bone and Schneiderian membrane rather than rotary instruments, with good results in terms of reduced surgical time, incidence of membrane perforation (4.3%), and other intraoperative complications [37].

The survival rate after maxillary sinus augmentation with a lateral window approach was 92.6%; it was lower than that of the displacement group (100%) and very similar to that registered for osteotome sinus floor elevation (OSFE; in the range of 87.5–98.7% between 2- and 3-year surveys) [29,30,31,38]. 

Peri-implant marginal bone losses after 3-year functional loading were 1.47 and 1.30 mm for the displacement and filling groups, respectively, and they appeared to be in line with the survival data registered by other authors. Radiological outcomes after OSFE showed an MBL within the range of 0.8–1.3 mm [35,36,39], whereas the peri-implant MBL after maxillary sinus augmentation with a lateral window approach ranged between 1.0 and 1.8 mm [30,40].

From the present point of view, a clinician who wants to plan an implant-supported fixed rehabilitation in the maxillary first/second premolar edentulous site (close to the anterior wall of the sinus floor) should consider the backward displacement of the anterior wall, giving the same results as traditional surgery, that is, sinus augmentation with a lateral window approach, but only over a limited area. The displacement produces a mean gain in the inclination of the anterior wall close to 30 degrees, so it intended to resolve the problem of hyperpneumatization when the angle between the pristine palatal vault and the occlusal cortical plate is at least equal to the amount of the gain (so, higher than 30°). This severely limited any possibility to enhance bone volume far from the bone plate. However, no xenogeneic bone material was used in the augmentation procedure, and a great amount of bone gain was obtained after 3 years, suggesting that the technique is highly reliable and successful.

Given the nature of the study, it should be noted that the presence of data regarding bone volume remodeling remains one of the most important criteria. On the other hand, the strength of the present study is the single brand and type of dental implant and the uniformity of surgical performances.

## 4. Conclusions

Together with the absence of any dental implant bulging, clinical and radiographic outcomes presented in the present study showed an effective bone gain around fixtures at a 3-year survey for sinus augmented by backward displacement of the anterior wall and sinus lifted with a lateral window approach and grafted with particulate bone. The displacement procedure seemed to have a higher success rate and led to a slightly higher marginal bone loss at the distal aspect than those of the conventional sinus lift technique.

## Figures and Tables

**Table 1:** Mean and standard deviation of volume at the preoperative (preop) stage and at the 3-year survey (3yrs) of the alveolar bone, and their difference, ΔBV (with %ΔBV as its percentage) from the preoperative stage to the 3-year survey (preop→3yrs) in volume of interest (VOI).

Times	BV_preop_		BV_3yrs_		Preop vs. 3Yrs
	Variable	Normality Test	Variable	Normality Test	*p*-Value
**displacement (cc)** ***n* = 23**	1.17 ± 0.34	0.8662 ^	1.53 ± 0.39	0.7716 ^	0.0017 *
**filling (cc)** ***n* = 20**	1.24 ± 0.41	0.4291 ^	1.94 ± 0.68	0.1907 ^	<0.0001 *
**displacement vs. filling** **(*p*-value)**	0.8076 °		**0.0352 °**		
**sample size** **(power 80%, α 0.05, β 0.2)**	878		54		
**Times**	**ΔBV (Preop→3 yrs)**		**%ΔBV (Preop→3yrs)**		**Angle of Displacement**
	**Variable**	**Normality** **Test**	**Variable**	**Normality** **Test**	**(Degrees)**
**displacement (cc)** ***n* = 23**	+0.36 ± 0.17	0.9983 ^	+34 ± 21	**0.0483 ^**	31 ± 6
**filling (cc)** ***n* = 20**	+0.71 ± 0.31	**0.0467 ^**	+57 ± 13	0.4135 ^	-
**distraction vs. grafting** **(*p*-value)**	**0.0001 °**		**0.0001 °**		
**sample size** **(power 80%, α 0.05, β 0.2)**	14		20		
**Times**	**MBL (Preop→3yrs)**				**Mesial vs. Distal**
	**Variable** **(Mesial)**	**Variable** **(Distal)**	**Variable** **(Mean)**	**Normality** **Test**	**(*p*-Value)**
**displacement (mm)** ***n* = 23**	1.29 ± 0.63	1.66 ± 0.72	1.47 ± 0.38	0.9710 ^	0.1350 *
**filling (mm)** ***n* = 20**	1.35 ± 0.67	1.25 ± 0.78	1.30 ± 0.58	0.9248 ^	0.6980 *
**displacement vs. filling** **(*p*-value)**	0.7977 °	**0.0497 °**	0.1026 °		
**sample size** **(power 80%, α 0.05, β 0.2)**	3572	102	250		

Marginal bone loss from the preoperative stage to the 3-year survey (preop→3yrs). Normal distribution test: ^ (Shapiro–Wilk test); statistical comparisons: * Wilcoxon signed-rank test assessing changes in time from the preoperative stage to the 3-year follow-up; ° Wilcoxon rank-sum test assessing changes between groups. Sample sizes calculated with a power of 80% and the probability of type I error of 5% obtained between the two groups for linear and volumetric outcomes.
